# Molecular and clinicopathologic characteristics of CNS embryonal tumors with *BRD4::LEUTX* fusion

**DOI:** 10.1186/s40478-024-01746-7

**Published:** 2024-03-18

**Authors:** Felipe Andreiuolo, Christina K. Ferrone, Sharika Rajan, Arie Perry, Ekin Guney, Elaine Cham, Caterina Giannini, Angus Toland, Nicholas Willard, Andrea Silveira de Souza, Karen Dazelle, Hye-Jung Chung, Omkar Singh, Kyle Conway, Nicholas Coley, Christopher Dampier, Zied Abdullaev, Drew Pratt, Patrick J. Cimino, Martha Quezado, Kenneth Aldape

**Affiliations:** 1 Department of Pathology, Rede D’Or, Rio de Janeiro, RJ Brazil; 2 D’Or Institute for Research and Education, Rio de Janeiro, RJ Brazil; 3 Department of Pathology, Instituto Estadual Do Cérebro Paulo Niemeyer, Rio de Janeiro, RJ Brazil; 4 Laboratory of Pathology, Center for Cancer Research, National Cancer Institute, National Institutes of Health, 10 Center Dr., Room 2S235, Bethesda, MD 20892 USA; 5 Department of Pathology, University of California, San Francisco, San Francisco, CA USA; 6 Department of Laboratory Medicine and Pathology, Mayo Clinic, Rochester, MN USA; 7 Department of Pathology, University of Colorado Hospital, Aurora, CO USA; 8 Department of Pathology, University of Michigan, Ann Arbor, MI USA; 9 Diagnostic Pathology Medical Group, Inc., Sacramento, CA USA; 10 Surgical Neurology Branch, National Institute of Neurological Disorders and Stroke, National Institutes of Health, Bethesda, MD USA

**Keywords:** Methylation, *BRD4::LEUTX*, Embryonal tumor

## Abstract

Central nervous system (CNS) embryonal tumors are a heterogeneous group of high-grade malignancies, and the increasing clinical use of methylation profiling and next-generation sequencing has led to the identification of molecularly distinct subtypes. One proposed tumor type, CNS tumor with *BRD4::LEUTX* fusion, has been described. As only a few CNS tumors with *BRD4::LEUTX* fusions have been described, we herein characterize a cohort of 9 such cases (4 new, 5 previously published) to further describe their clinicopathologic and molecular features. We demonstrate that CNS embryonal tumor with *BRD4::LEUTX* fusion comprises a well-defined methylation class/cluster. We find that patients are young (4 years or younger), with large tumors at variable locations, and frequently with evidence of leptomeningeal/cerebrospinal fluid (CSF) dissemination. Histologically, tumors were highly cellular with high-grade embryonal features. Immunohistochemically, 5/5 cases showed synaptophysin and 4/5 showed OLIG2 expression, thus overlapping with CNS neuroblastoma, *FOXR2*-activated. DNA copy number profiles were generally flat; however, two tumors had chromosome 1q gains. No recurring genomic changes, besides the presence of the fusion, were found. The *LEUTX* portion of the fusion transcript was constant in all cases assessed, while the *BRD4* portion varied but included a domain with proto-oncogenic activity in all cases. Two patients with clinical follow up available had tumors with excellent response to chemotherapy. Two of our patients were alive without evidence of recurrence or progression after gross total resection and chemotherapy at 16 and 33 months. One patient relapsed, and the last of our four patients died of disease one month after diagnosis. Overall, this case series provides additional evidence for this as a distinct tumor type defined by the presence of a specific fusion as well as a distinct DNA methylation signature. Studies on larger series are required to further characterize these tumors.

**Supplementary Information:**

The online version contains supplementary material available at 10.1186/s40478-024-01746-7.

## Introduction

DNA methylation arrays greatly improve diagnostic precision and reliability, and are highly effective for the discovery of new tumor types [1]. This is particularly true of central nervous system (CNS) embryonal tumors, a tumor class comprising a growing list of subgroups largely refined by epigenetic studies [2]. These tumors are high-grade malignancies composed of primitive neuroepithelial cells, predominantly seen in childhood. The 2021 update to the World Health Organization (WHO) classification of CNS tumors saw the incorporation of several new CNS embryonal tumors subtypes, including: cribriform neuroepithelial tumor; CNS neuroblastoma, *FOXR2*-activated; and CNS tumor with BCOR internal tandem duplication [3].

Recently, two cases of CNS embryonal tumors harboring a *BRD4::LEUTX* fusion were described [4, 5]. One of these reports characterized the fusion in a large series of pediatric cancers [5], while the other incorporated detailed clinical and pathological data, including methylome analysis matching to the novel Heidelberg version 12 classifier class “CNS embryonal tumor with *BRD4::LEUTX* fusion” [4]. Three additional cases also exist in the literature, which while not specifically described as such, were initially presented before the version 12 of the Heidelberg classifier was available to interrogate this provisional tumor type [6–8]. In addition to these 5 cases, we describe 4 additional unpublished new cases of CNS tumors with *BRD4::LEUTX* fusions, thereby expanding the limited characterization available for this tumor type to date.

## Methods

### Tissue samples and datasets

The use of human subject material was performed in accordance with the World Medical Association Declaration of Helsinki and with the approval of the participating Institutional Review Boards. This study included previously described cases of CNS embryonal tumors with *BRD4::LEUTX* fusion as well as cases from the Laboratory of Pathology clinical consult service at the National Cancer Institute (NCI) in Bethesda, MD, USA, as well as available methylation array data in the form of raw IDAT files. Referral consult cases were received from the following institutions: Mayo Clinic (Rochester, Minnesota); UCSF Benioff Children’s Hospital (Oakland, California); Children’s Hospital Colorado (Aurora, Colorado); and University of California San Francisco Medical Center (San Francisco, California). Each of the referral consult cases was reviewed for histopathology as part of the diagnostic process by at least two board-certified neuropathologists (PJC, MQ, DP, KA). Results of IHC stains were retrieved from the reports from each center as available.

### Nucleic acid extraction and next-generation sequencing

Genomic DNA and RNA was extracted from 5 μm sections of formalin fixed paraffin embedded tissue (FFPE) sections mounted on slides using the QIAamp DNA FFPE Tissue Kit (Qiagen, Hilden, Germany), RNeasy FFPE Tissue Kit (Qiagen), or AllPrep DNA/RNA FFPE Kit (Qiagen). DNA was set aside for methylation analysis as described below. Next-generation sequencing (NGS) was performed using commercial TruSeq RNA Exome panel (Illumina). Exome RNA sequencing libraries were prepared with 100 ng tumor RNA using the Illumina RNA Prep with Enrichment (L) Tagmentation kit (Illumina) with Exome Probe Panel (Illumina). Final enriched libraries are sequenced on NextSeq 550DX or NovaSeq 6000 (Illumina).

### Data processing and variant analysis

After sequencing, the FASTQ files were aligned to human reference genome hg19 (GRCh37) using STAR aligner [9] to generate BAM files. The resulting BAM files were used by Arriba tool [10] to predict fusion calls. The filtered fusions (VCF file) are uploaded to the QIAGEN Clinical Insight (QCI; Qiagen) for annotation, classification, and interpretation. All variants were manually reviewed by visualizing the raw sequencing read alignments using the Integrative Genomics Viewer [11].

### DNA methylation profiling and initial analysis

Samples were processed as previously described [12]. Briefly, genomic DNA (250 ng as the standard) was bisulfite-converted (EZ DNA Methylation Kit, Zymo Research D5001). When the total amount of DNA available was less than 250 ng, the sample was run as well. Bisulfite-converted FFPE DNA was processed with the Infinium FFPE DNA Restore kit (Illumina, USA) and was assayed on Infinium MethylationEPIC kit (Illumina, USA), according to the Infinium HD FFPE Methylation Assay automated protocol (Illumina, USA). Methylation data was processed using versions 11.b6 and 12.b6 of the DKFZ-Heidelberg classifiers and NCI-Bethesda classifier [6]. Copy number variant (CNV) profiles were inferred using the R “conumee” package (http://bioconductor.org/packages/conumee/) as implemented in the classifier package. Tumor purity was estimated using RF_purify package and LUMP score from methylation profile [13, 14].

## Results

### Case descriptions

Five previously published cases [4–8] were identified in the following manner. One case presented a dedicated case report for a tumor matching to the methylation class “CNS embryonal tumor with *BRD4::LEUTX* fusion”, with demonstration of the fusion [4]. A second case had been discovered in a large series of pediatric cancers where the *BRD4::LEUTX* fusion had been identified in one case of the series [5]. A third case was found in a series of medulloblastoma, where re-evaluation using version 12 of the Heidelberg classifier showed a match to CNS embryonal tumor with *BRD4::LEUTX* fusion [7]. A fourth case was included as a low-score match to group 3 medulloblastoma in the initial paper describing the Heidelberg version 11 methylation classifier [6], but which in our analysis of the Heidelberg version 12 classifier, matched to CNS embryonal tumor with *BRD4::LEUTX* fusion. The fifth case was described in a series of *FOXR2*-activated neuroblastomas, as a potential diagnostic mimic [8]. In addition to these 5 cases, 4 cases were identified in the clinical diagnostic methylation service at the National Institutes for Health. Clinical and molecular features for these 9 cases are summarized in Table 1. All patients for whom age was known were young children (up to 4 years) with large masses; three female, two male, and four of unknown sex. The initial (pre-methylation profiling) diagnosis, when known, suggested a CNS embryonal tumor, either NOS or a CNS neuroblastoma, *FOXR2*-activated.

**Table 1:** Clinical and molecular features of embryonal tumors with *BRD4-LEUTX* fusion

Reference case	Age at diagnosis (years), sex	Initial diagnosis	Tumor location	Methylation based classification (v12.b6); score	Fusion (BRD4::LEUTX)
1 BB27	1.4, F	Embryonal tumor, favor CNS neuroblastoma *FOXR2*-activated	Cerebral peduncle/thalamus	CNS Embryonal tumor with BRD4:LEUTX fusion 1.0	Exons 11:2
2 AI01	3, M	CNS Embryonal tumor NOS (grade 4)	Interpeduncular	CNS Embryonal tumor with BRD4:LEUTX fusion 1.0	Exons 14:2
3 DQ95	1.25, M	Embryonal tumor, favor CNS neuroblastoma *FOXR2*-activated	Large cystic/solid thalamus	CNS Embryonal tumor with BRD4:LEUTX fusion 0.98	Exons 11:2
4 EV64	1, F	Embryonal tumor; Pineoblastoma?	Tectal plate/ third ventricle	CNS Embryonal tumor with BRD4:LEUTX fusion 1.0	Exons 11:2
5 Lebrun	4, F	Embryonal tumor (NOS)	Left parietal	CNS Embryonal tumor with BRD4:LEUTX fusion 0.99	Exons 13:2
6 Wong	Infant, NA	Embryonal CNS tumor	NA	NA	Exons 11:2
7 Northcott (case: SJPB18)	NA	Medulloblastoma	NA; Posterior fossa?	CNS Embryonal tumor with BRD4:LEUTX fusion 0.99	NA
8 Capper (case: DIA_0871)	NA	NA	NA	CNS Embryonal tumor with BRD4:LEUTX fusion 0.79	NA
9 Tauziede-Espariat (case 3)	1, M	CNS neuroblastoma *FOXR2*-activated	Left frontoparietal	CNS Embryonal tumor with BRD4:LEUTX fusion 0.99	Exons 11:2

*NA* not available

### Histopathologic and immunohistochemical tumor characterization

**Fig. 1:**
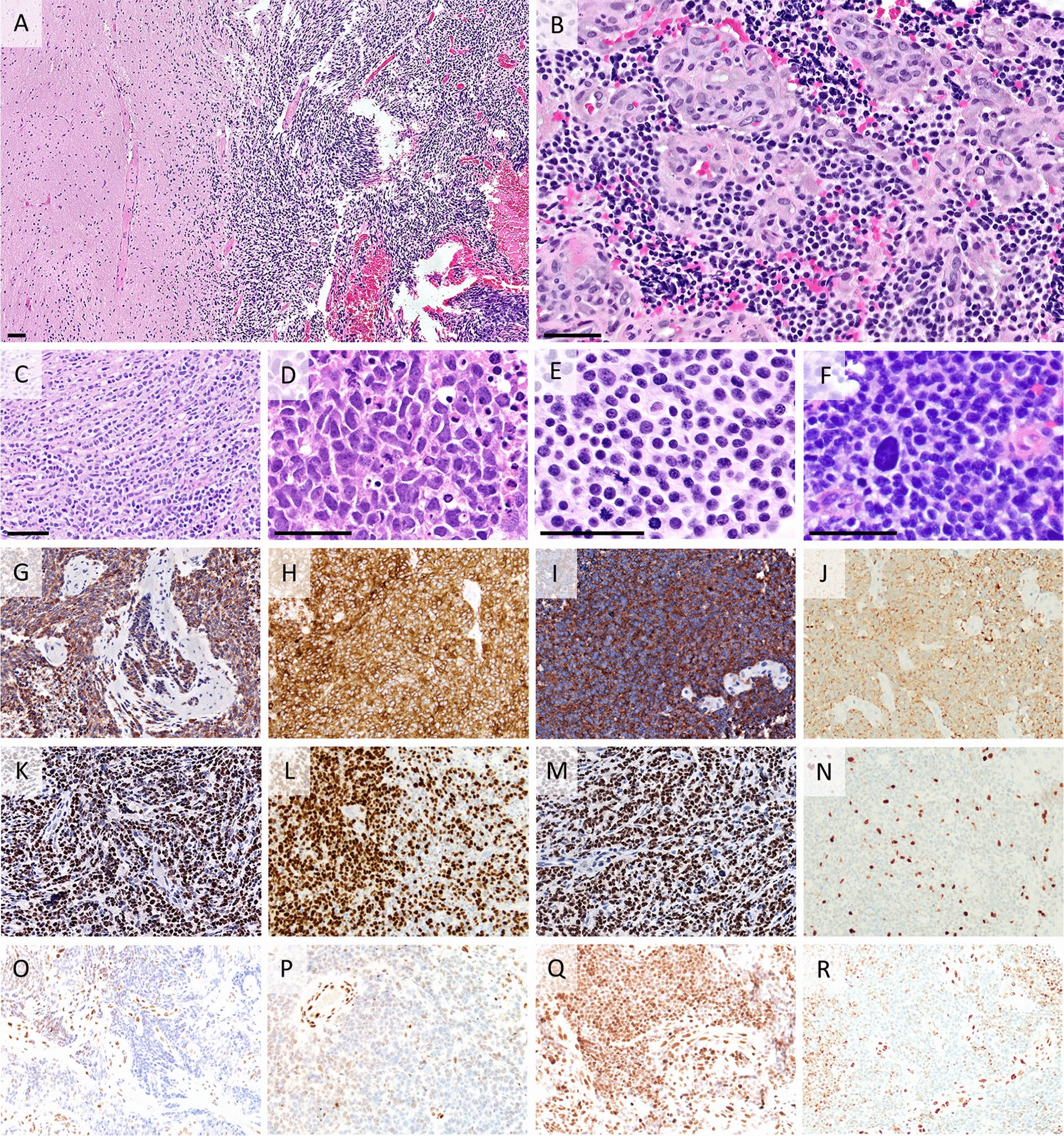
Histology and Immunohistochemistry. All scale bars measure approximately 50 µm. Immunostains were originally photographed at 20 × 10 = 200X magnification, with some images resized slightly to fit within the figure. **A** Case #3 was relatively well-circumscribed from adjacent nervous tissue. **B** Case #1 showed small monomorphic cells with round hyperchromatic nuclei and scant cytoplasm. Abundant proliferated vessels with particularly thick walls were seen at least focally in all cases. **C** Case #1: prominent cell streaming pattern. **D** Case #2 featured more irregularly shaped nuclei. **E** All cases had prominent mitotic activity as seen in case #3. **F** Case #4: An enlarged atypical nucleus stands out against small, regular nuclei. **G–J** Diffuse positivity of immunohistochemistry (IHC) for synaptophysin in cases #1 (**G**), #2 (**H**), #3 (**I**) and #4 (**J**). **K**–**N**. Expression of OLIG2 positivity seen in all cases, at variable levels. **K** Case #1 showed strong and diffuse positivity. **L.** Most cells were positive in case #2. **M** Case #3 displayed strong and diffuse positivity. **N** Case #4 showed scant OLIG2 positive cells. **O**–**R** H3K27me3 IHC for cases #1 (**O**), #2 (**P**), #3 (**Q**) and #4 (**R**). **Q** Case #3 showed retained expression. **R** Case #4 showed loss of expression in tumors cells with a mosaic pattern

Histologic and immunohistochemical features from our cases (#1–4), along with data retrieved from two previously published cases are described here. Histologically, all six of the assessed cases were highly cellular, showing embryonal morphology with diffuse arrangement (Fig. 1 and Additional file 1: Table S1), with a cell-streaming pattern seen in two cases, and Homer–Wright rosettes in one case. In two cases, the borders with brain parenchyma could be histologically assessed, showing sharp tumor circumscription. Neoplastic cells were small with round to oval nuclei, with two cases presenting some more irregularly shaped nuclei. Cytoplasm was scant. Scattered large, bizarre nuclei were seen in three cases. Four cases displayed necrosis. Brisk mitotic activity and apoptosis were seen in all samples. All cases displayed microvascular proliferation, often with marked multilayering of vessel walls. Calcifications were detected in two tumors histologically, and suspected in a third tumor by imaging exams, but could not be confirmed on the small biopsy examined. All tumors expressed synaptophysin (diffusely), and five expressed OLIG2 variably (Fig. 1). GFAP and neurofilament were expressed in a subset of tumor cells in 2/5 cases examined. Interestingly, one tumor had complete loss of H3k27me3 expression in tumor cells while three tumors showed a markedly attenuated expression of H3k27me3 in tumor cells with areas showing clear loss (Fig. 1) and areas with weak expression in a mosaic configuration. In all three cases tested, immunohistochemistry (IHC) was negative for the mutant protein H3 p.K27M. INI1 and BRG1 proteins were expressed in 5/5 tumors and LIN28 was negative in 3/3 tested. EZHIP was negative for 1/1 cases tested. We note a number of CNS tumor types where H3K27me3 expression is described as lost in specific cases, including IDH-mutant gliomas [15]). Results for all IHC performed are listed in Additional file 1: Table S2.

### Methylation, NGS, and CNV findings

**Fig. 2:**
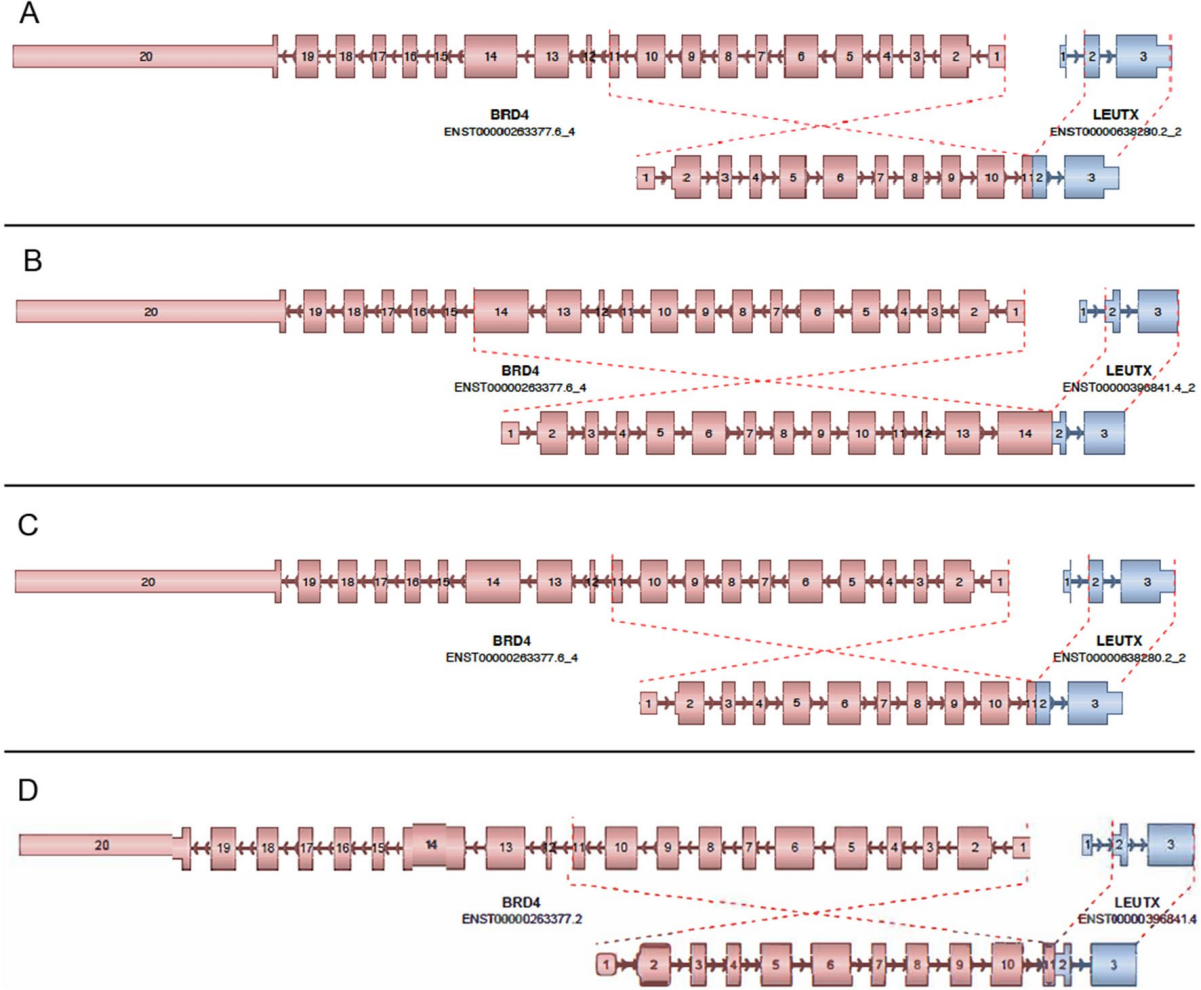
Arriba fusion diagrams. **A** Case #1; fusion breakpoints for *BRD4* and *LEUTX*: chr19:15364963 and chr19:40275167. **B** Case #2; fusion breakpoints for *BRD4* and *LEUTX*: chr19:15353711 and chr19:40275167. **C** Case #3; fusion breakpoints for *BRD4* and *LEUTX*: chr19:15364963 and chr19:40275167. **D** Case #4; fusion breakpoints for *BRD4* and *LEUTX*: chr19:15364963 and chr19:40275167

**Fig. 3:**
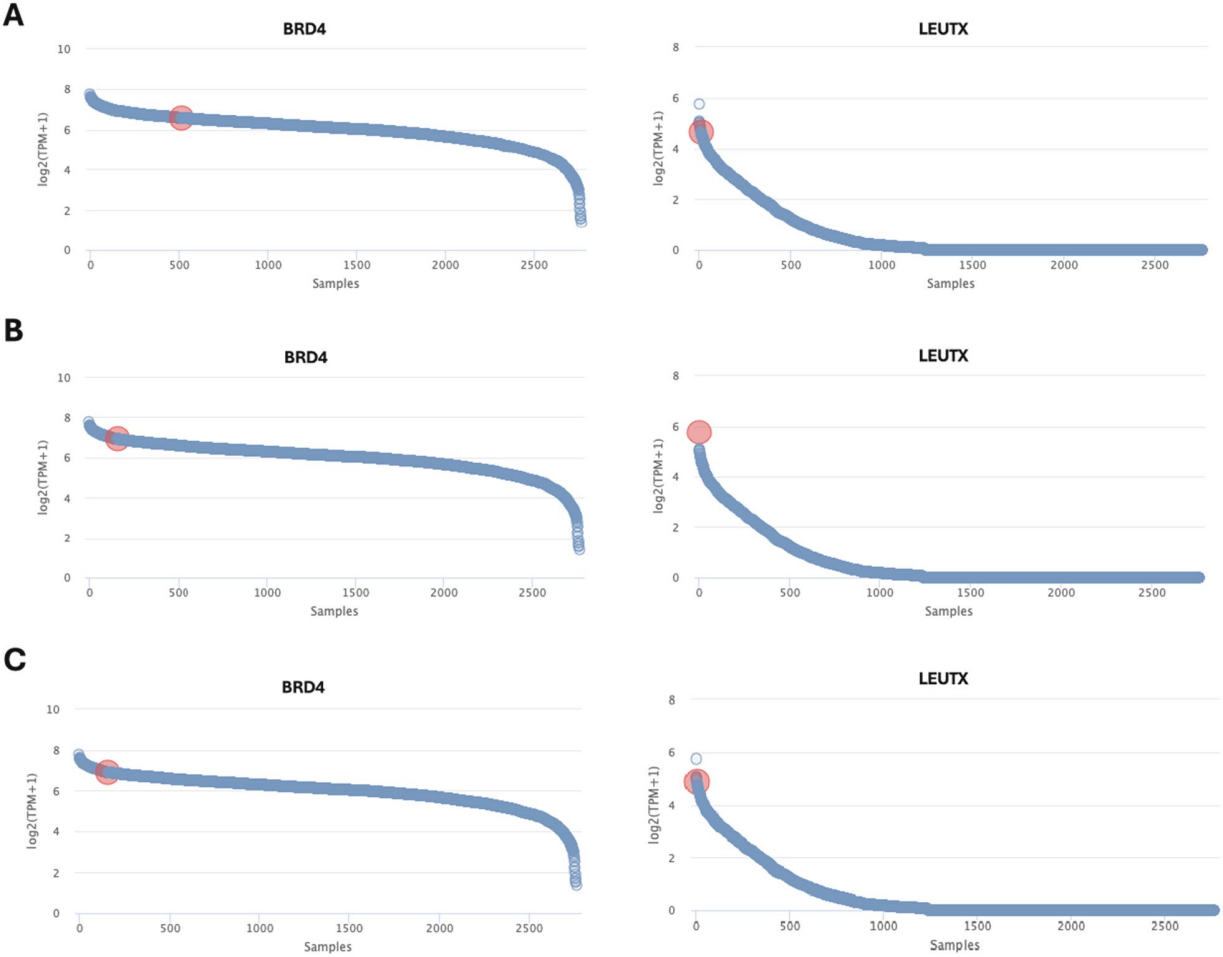
Expression of *BRD4* and *LEUTX*. Available for cases #1–3. TPM = transcripts per kilobase million; i.e. counts per length of transcript (kb) per million reads mapped. Total samples included in clinical cohort for expression comparison = 2765. **A** Case #1; *BRD4* TPM = 6.57, *LEUTX* TPM = 4.66. **B** Case #2; *BRD4* TPM = 6.91. *LEUTX* TPM = 5.74 **C** Case #3; *BRD4* TPM = 6.91, *LEUTX* TPM = 4.87

**Fig. 4:**
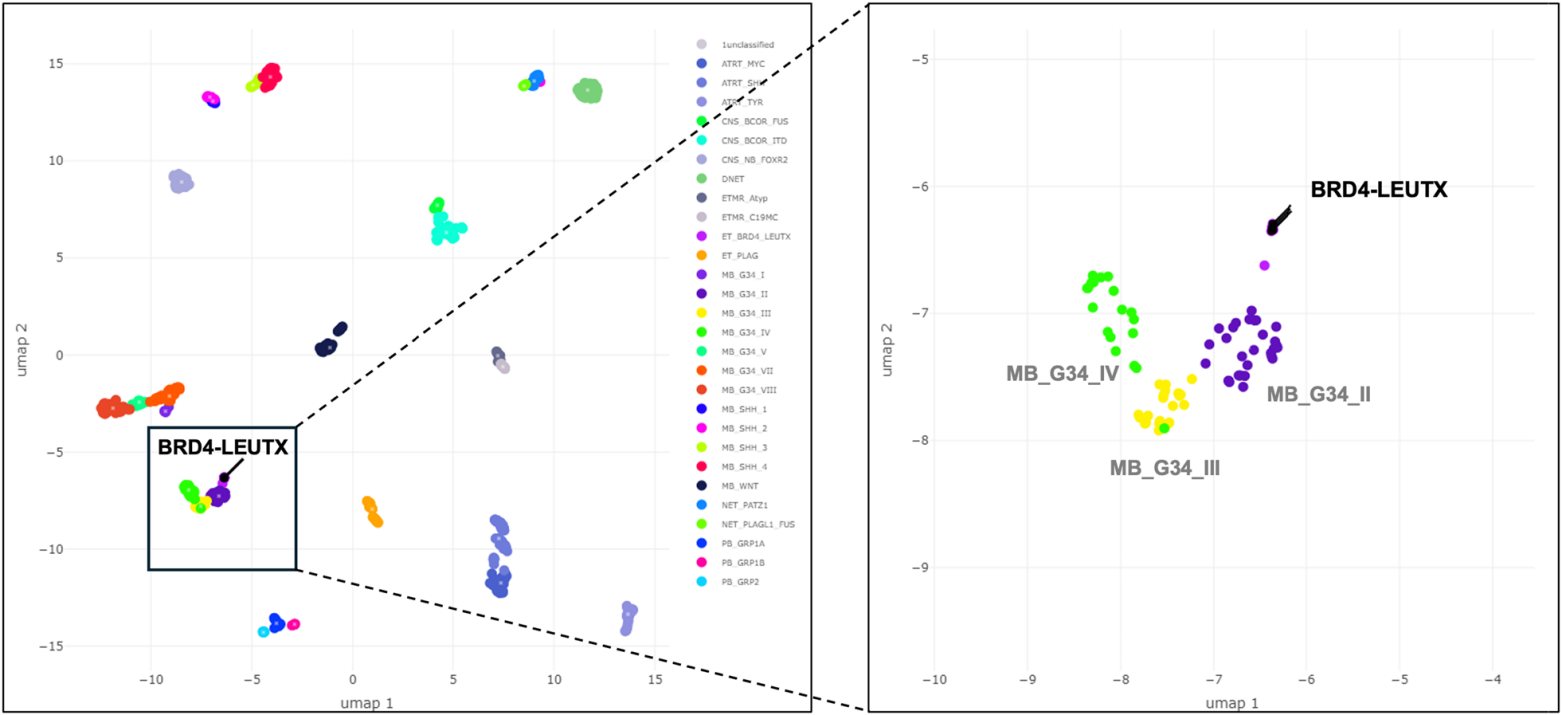
UMAP diagrams showing distinct cluster for CNS Embryonal Tumor with *BRD4::LEUTX* fusion. Diagram includes the 6 cases for which IDAT files were available: #1–4, #7, and #8. Left panel shows cases in relation to a variety of embryonal tumor types. Right panel shows close-up, where nearby clusters include group 3 medulloblastomas (MB_G34_II; MB_G34_III; MB_G34_IV). Abbreviations are as per DKFZ version 12.8 methylation class names (https://www.molecularneuropathology.org/mnp/classifiers/14)

The DKFZ-Heidelberg classifier version 12.5 (and its subsequent iterations) included a methylation class “CNS embryonal tumour with *BRD4*:*LEUTX* fusion” which allowed for identification of tumors that matched to this class. Methylation profiling of our 4 cases, as well as 4 cases culled from the literature matched to this class. Seven of these 8 cases reached the 0.84 confidence threshold in the match, with the 8th case showing a ‘suggestive’ score (0.79) for this class (Table 1). RNA sequencing was performed for the 4 NIH cases and 3/5 outside cases, identifying the presence of the *BRD4::LEUTX* fusion for each of the 7 cases interrogated. All fusion junctions involved exon 2 of *LEUTX*, retaining the homeobox domain, while the *BRD4* component of the fusions involved exons 11, 13, or 14, maintaining the bromodomain in all cases (Table 1 and Fig. 2). Inspection of the DNA copy number changes did not show recurrent alterations (copy number profiles for the 4 NIH cases are shown in Additional file 2: Fig. S1), and in addition copy number breakpoints at either the *BRD4* or *LEUTX* loci were not a feature of this neoplasm. Gene expression was available for 3 of the 4 cases profiled at the NIH, where the expression of *LEUTX* and *BRD4* was compared to the clinical dataset of > 2700 samples of a variety of tumor types with gene expression data available. In all 3 cases, expression of *BRD4*, and especially that of *LEUTX* was high relative to expression of these genes in the broad dataset of tumor types (Fig. 3). The elevated expression of *LEUTX* seen in these tumors is in line with a prior study that reported a *BRD4::LEUTX* fusion in a pediatric sarcoma [16]. Inspection of methylation signatures using UMAP for the four NIH cases and 2 of the previously published cases fore which (IDAT) files were available showed that CNS tumors with *BRD4::LEUTX* fusion cluster together, near but separate from group 3 medulloblastoma (Fig. 4). Lastly, DNA sequencing did not identify any recurrent pathogenic or likely pathogenic mutations in the 3 cases (cases 1–3) that were sequenced on a large (~ 500 gene) panel.

### Radiological findings and clinical follow-up

**Fig. 5:**
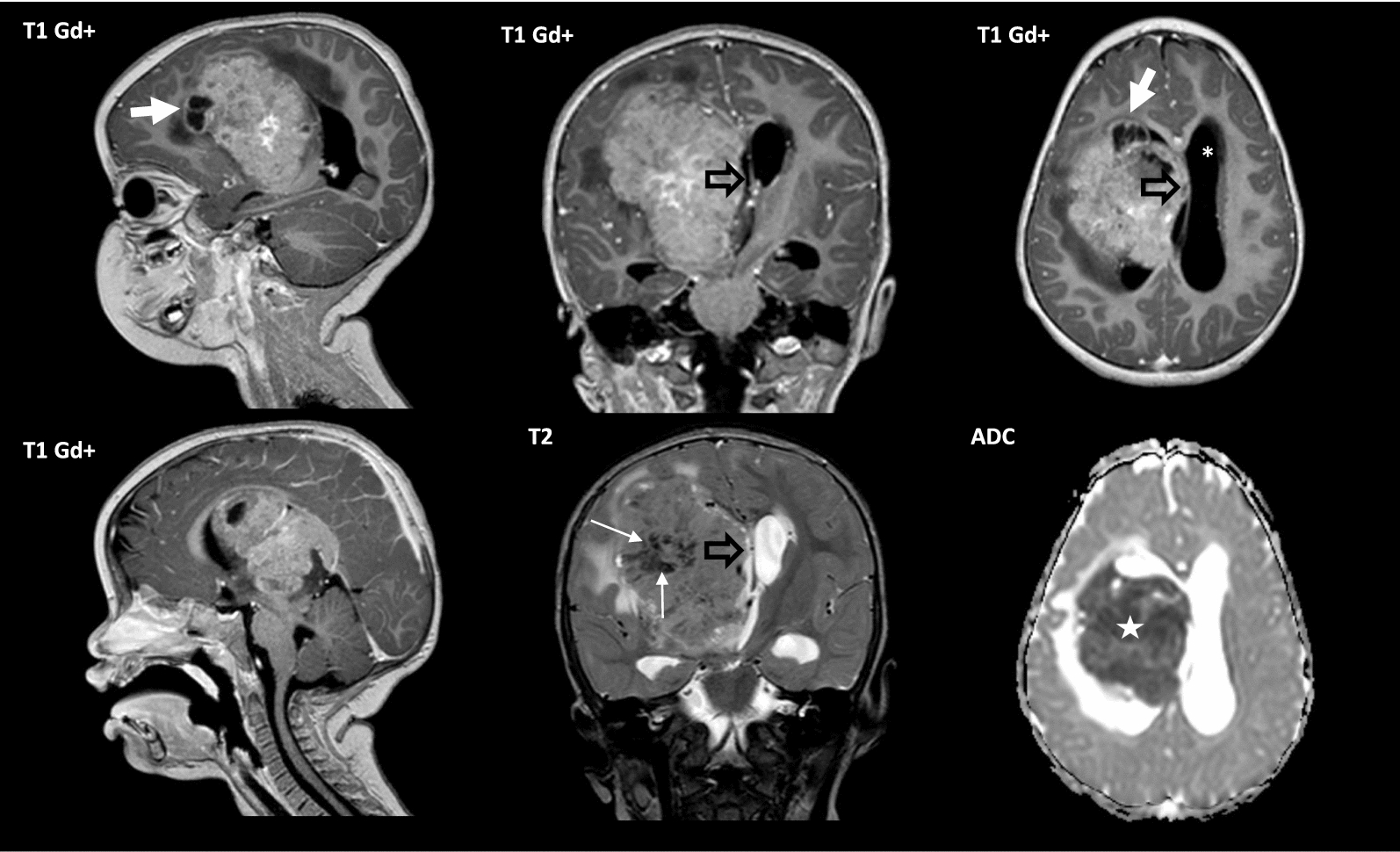
Imaging features of tumors with *BRD4::LEUTX* fusion. Case #3 shown: Large right supratentorial brain tumor, mostly solid, with associated mass effect, midline shift (open black arrow) and hydrocephalus (white asterisk). Intense heterogeneous contrast enhancement, cystic change (thick white arrow), marked restriction on ADC map (white star) and hemosiderin deposition (thin white arrow) are also observed

**Fig. 6:**
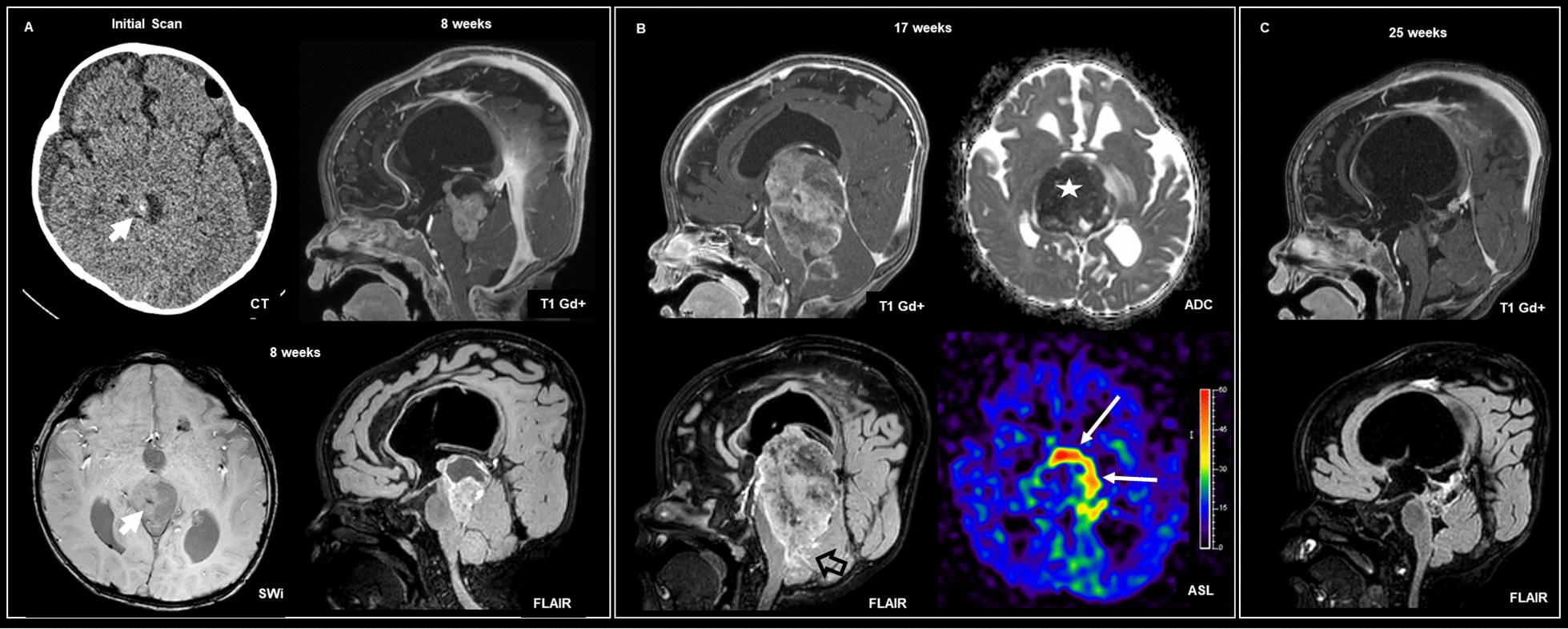
Radiological findings demonstrating response to chemotherapy. Case #4 shown. **A** A small foci of calcification is depicted on CT scan and susceptibility-weighted imaging MRI (short white arrow). Supratentorial obstructive hydrocephalus and macrocrania are also observed. **A**, **B** Heterogeneous tectal and pineal region solid cystic mass, with intense heterogeneous enhancement and rapid overgrowth within two months. **B** The solid component is highly cellular with marked restriction on ADC map (white star). A rim of increased blood flow on ASL perfusion map is observed in the periphery of the mass (long white arrows), possibly related to neovascularization. Leptomeningeal enhancement is also observed (open black arrow). **C** Significant shrinkage of the tectal and pineal region mass after chemotherapy was observed, with nearly complete resolution of the intracranial leptomeningeal enhancement

A representative series of images are shown for case #3, showing a large tumor with heterogeneous contrast enhancement, cystic change and mass effect (Fig. 5). With respect to patient follow-up, cases #1 (15-month-old female) and #2 (3-year-old male) were both alive without evidence of recurrence or progression at with near total resection followed by chemotherapy. Case #1 was alive one year post-therapy. Case #3 was a 14-month-old male who underwent a stereotaxic biopsy followed by four cycles of chemotherapy, with marked reduction on the enhancing solid tumor component, and gross total resection four months after the initial diagnosis. Two weeks post-surgery, there were signs of local recurrence and salvage therapy was indicated. Case #4 was a 12-month-old female with hydrocephalus with a heterogeneously enhancing mass with evidence of leptomeningeal spread. The patient underwent a stereotactic biopsy and the tumor showed striking response to chemotherapy with tumor shrinkage and nearly complete resolution of the intracranial leptomeningeal enhancement after chemotherapy (Fig. 6). Of note, cases #1–4 were all described to have been treated in a manner similar to clinical trial NCT00336024, where the chemotherapy regimen included cisplatin, cyclophosphamide, vincristine, and etoposide followed by thiotepa. Case #5 was a 4-year female [4] presenting with a large left parietal mass with multiple smaller supra- and infra- tentorial tumor nodules, as well as signs of subependymal and leptomeningeal dissemination. The patient died of disease shortly after surgery.

## Discussion

We report on a series of cases that show fusions involving *BRD4* and *LEUTX* and match to the corresponding DKFZ-Heidelberg class describing this tumor type. Patients with these tumors are young, and the tumors show features of an embryonal neoplasm. These are rare tumors, but important to recognize, as they are genomically and epigenetically distinct from other CNS embryonal neoplasms. The *BRD4* gene plays a crucial role in transcriptional regulation and in development, it maintains stem cell pluripotency. Dysregulation of *BRD4* has been linked to various cancers, where it contributes to cell proliferation and tumor progression. Known fusions include the *BRD4::NUT* fusion in NUT midline carcinoma [17–19]. The *LEUTX* gene (also known as Leucine Twenty Homeobox), is a homeobox gene with a role in embryonic development and in regulating early cell differentiation. Fusions in this gene also play a role in cancer, including in primary CNS sarcomas involving *CIC::LEUTX* fusions [20, 21], and of interest, *CIC::LEUTX* fusions have recently been described in a novel glioneuronal tumor [22].

Interestingly, all fusions had the same breakpoint in *LEUTX* (exon 2; Fig. 2), which has previously been shown to lead to increased *LEUTX* expression, otherwise present almost exclusively in non-neoplastic embryonal tissues [16]. In three cases (#1, #3, and #4) the fusion transcript involving exon 11 of *BRD4* was identical to the one reported in an embryonal CNS tumor and a pediatric sarcoma with epithelioid features [16]. The *BRD4* portion encoded by this transcript (in the estimated chimeric protein) has the same length and structure of a shorter isoform of the protein (BRD4-S) previously shown to be implicated in breast cancer growth and metastasis, potentially mimicking BRD4-S pro-oncogenic activity [23, 24]. In case #2 and case #5 [4], the *BRD4* component of the fusion transcript is longer (Table 1) but still does not include a positive transcription elongation factor b (P-TEFb) interaction domain, and the intrinsically disordered region (IDR) is shorter than in the long *BRD4* isoform (BRD4-L); the latter being associated with tumor suppressor activity [25]. Of note, small-molecule BRD4 inhibitors and BRD4 degraders have shown promising results in preliminary studies in hematological and solid malignancies, and may represent a future therapeutic avenue for these aggressive tumors [26].

Despite molecular similarity, morphological and immunohistochemical features do not appear to overlap between *BRD4::LEUTX* fused CNS tumors and the single case of sarcoma with epithelioid features reported in the literature [16], nor with one case of possible alveolar rhabdomyosarcoma also reported to harbor a *BRD4::LEUTX* fusion [27]. DNA methylation profiling of these sarcomas would be helpful to determine how epigenetically similar these diseases are to CNS embryonal tumors with the same fusion. Indeed, pathological features can overlap between embryonal tumor subtypes, particularly those with *BRD4::LEUTX* fusions and *FOXR2*-activated neuroblastomas; most notably the small poorly differentiated cells, abundant necrosis, and co-expression of immunomarkers synaptophysin and OLIG2. A further overlapping feature with *FOXR2-*activated neuroblastoma is the gain of chr1q, which was found in two of our cases, and positivity for SOX10 seen in one of two cases tested [7].

Depending on tumor location, other embryonal tumors such as medulloblastoma and pineoblastoma should be considered in the differential diagnosis for this tumor type. Well-formed rosettes were not seen in all cases in our series, but these are not necessarily observed in medulloblastomas and pineoblastomas. OLIG2 positivity would be unexpected in both tumors. Embryonal tumors with multilayered rosettes (ETMRs) which otherwise lack rosettes, a medulloepithelioma component, and neurocytic/neuropil-rich areas could also resemble embryonal tumors with *BRD4::LEUTX* fusions, but they are generally negative for OLIG2 and positive for LIN28A. The recently described entity “CNS tumor with *PLAGL* amplification” (particularly *PLAGL2* in infants and toddlers) also shows overlapping embryonal features with the tumors in this series [28], nonetheless with a different immune-profile—OLIG2 being mostly negative, and synaptophysin being patchy and weak. Interestingly, reduction of H3k27me3 was detected in three cases, one in the tectal plate/third ventricle, and two located in peduncular/thalamic region, with complete loss of expression reported in one case in the parietal lobe [3]. Nonetheless, these potential mimics of diffuse midline glioma (DMG), K27-altered tumors seem to be more circumscribed, without evidence of diffuse infiltration both radiologically and histologically. Also, most DMG show glial morphology, and most do not exhibit extensive positivity for synaptophysin. Moreover, the loss of H3k27me3 can be observed in different CNS tumors and is caused by various mechanisms, generally leading to loss of transcriptional repression [29].

Our sample size is small, but initial clinical investigation showed that these were clinically aggressive, and the evolution of patient #5, who died of disease progression shortly after surgery, is in line with these features. Nevertheless, two patients who underwent stereotactic biopsies showed tumor reduction with chemotherapy, permitting subsequent gross total resection, though one of these relapsed. Two patients were alive without evidence of recurrence or progression after gross total resection and chemotherapy 16 and 33 months after initial diagnosis, one of them already one year off-therapy, suggesting that at least some of these tumors may benefit from chemotherapy.

## Conclusions

In this study, we find that CNS embryonal tumor with *BRD4::LEUTX* fusion is a provisional tumor type occurring in young children. They comprise a well-defined methylation cluster separate from other embryonal tumors and should therefore be considered in young children with small round cell tumors that do not match any known tumor classes; most tumors are synaptophysin and OLIG2 positive. Some of these tumors may show response to chemotherapy regimens. Of note, BRD4 is a bromodomain protein that plays a key role in epigenetic memory, and bromodomain inhibitors have been suggested as a possible therapeutic strategy in other tumor types. Studies on larger cohorts are required to further characterize these tumors.

### Supplementary Information

**Additional file 1: Table S1.** Main histopathologic findings in tumors with *BRD4*::*LEUTX* fusion. **Table S2.** Main immunohistochemical findings in tumors with *BRD4*::*LEUTX* fusion.

**Additional file 2: Fig. S1.** CNV profiles. A. Case #1; unremarkable. B. Case #2; alterations include gain of 1q21.1q44, loss of 14q23.3q32.33 (including *DICER1* and *AKT1*), and gain of chromosome 19 with a focal loss of 19q13.33q13.43 (including *PPP2R1A*, *CACNG6*, *C19MC* and *TTYH1*). C. Case #3; unremarkable. D. Case #4; unremarkable.

## Data Availability

Primary data will be provided upon reasonable request.

## Abbreviations

BAM: Binary alignment map
*BRD4::LEUTX*: Bromodomain containing 4-leucine twenty homeobox
CNS: Central nervous system
CNV: Copy number variant
CSF: Cerebrospinal fluid
DKFZ: Deutsches Krebsforschungszentrum
DMG: Diffuse midline glioma
ETMR: Embryonal tumor with multilayered rosettes
FASTQ: FAST quality
FFPE: Formalin-fixed paraffin-embedded
*FOXR2*: Forkhead box R2
GFAP: Glial fibrillary acidic protein
GTR: Gross total resection
H3k27me3: Tri-methylation measure of lysine 27 on histone 3 protein
H3K27M: Lysine 27 mutated to methionine in histone 3 gene isoforms
IDAT: Intensity data
IDR: Intrinsically disordered region
IHC: Immunohistochemistry
NGS: Next-generation sequencing
OLIG2: Oligodendrocyte transcription factor 2
P-TEFb: Positive transcription elongation factor b
VCF: Variant call format
