# Supplementary figures and images for: Molecular and clinicopathologic characteristics of CNS embryonal tumors with BRD4::LEUTX fusion
Source: Acta Neuropathol Commun. 2024 Mar 18;12:42. doi: 10.1186/s40478-024-01746-7 (PMC10946093; doi:10.1186/s40478-024-01746-7)

**A**

**
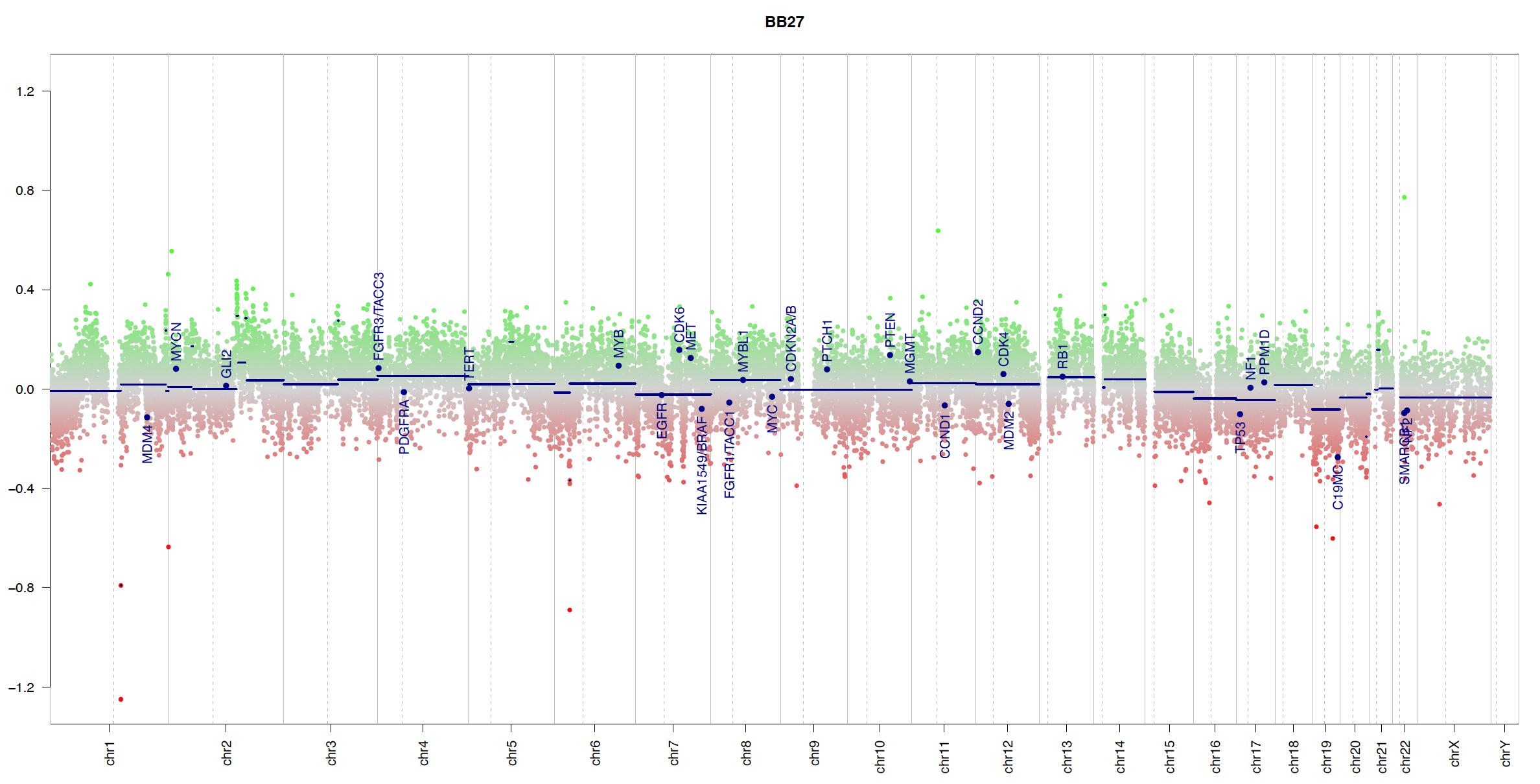
**

**B**

**
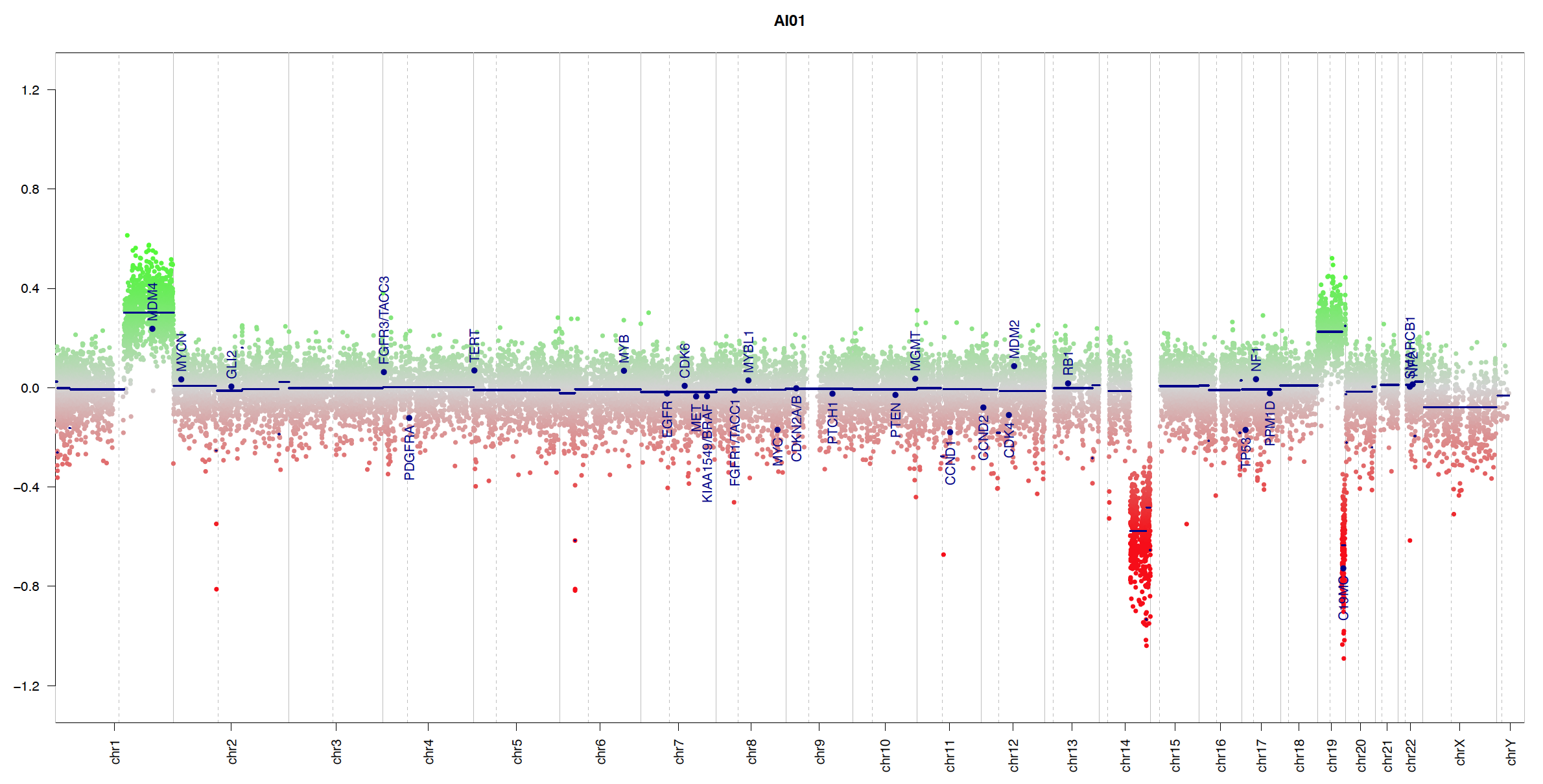
**

**C**

**
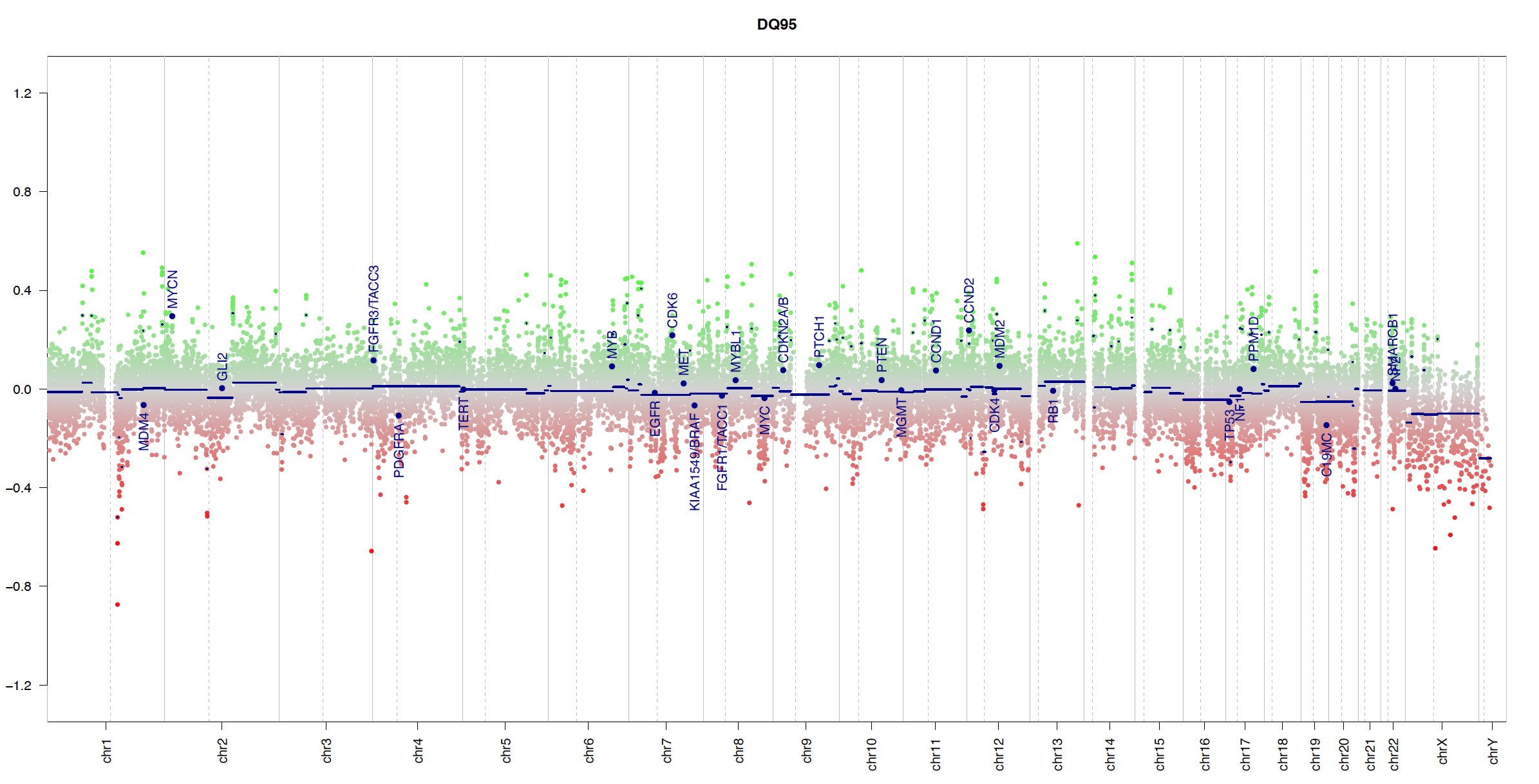
**

**D**


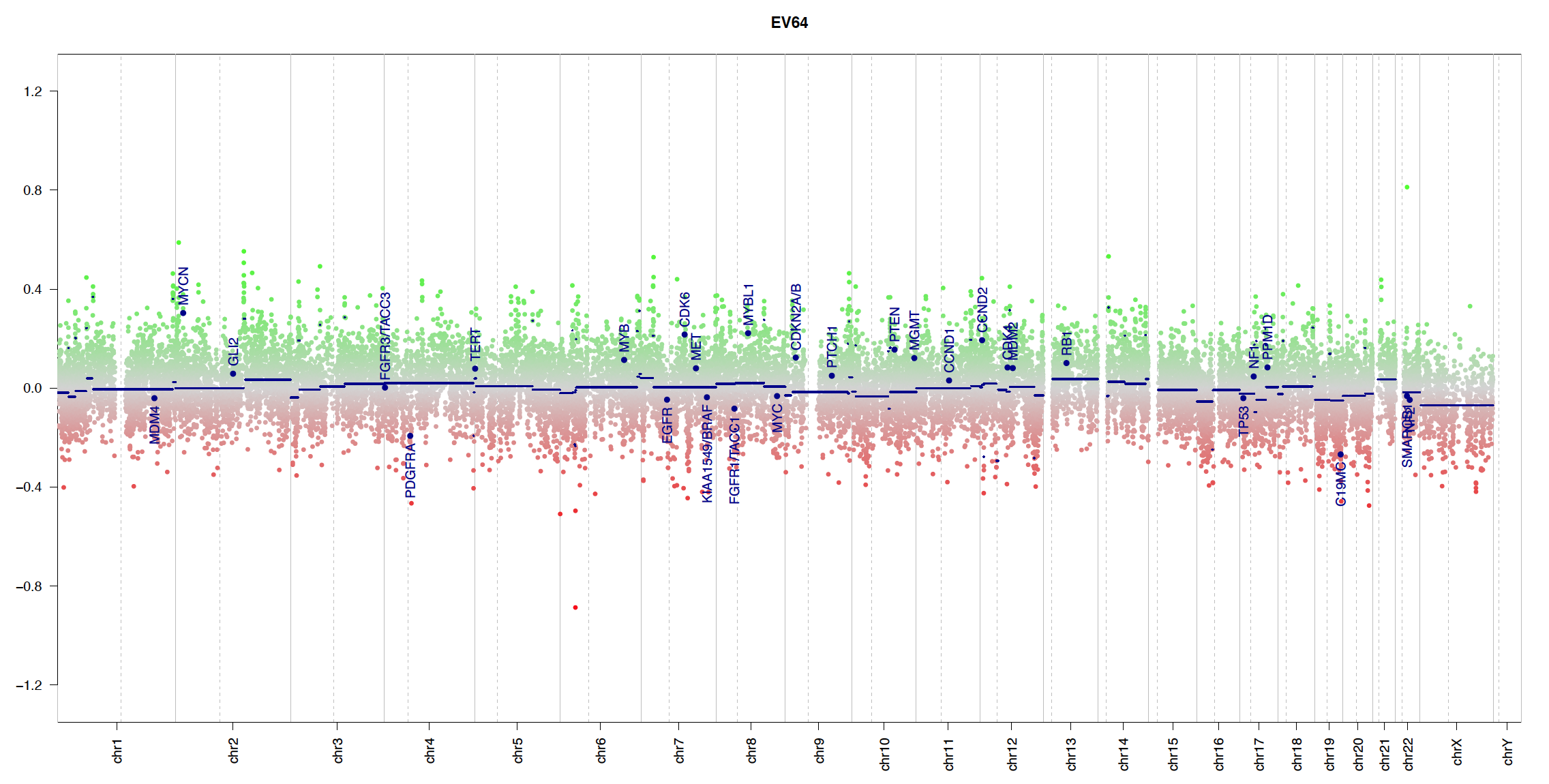

Supplement: Supplementary file 2 — Additional file 2: Fig. S1. CNV profiles. A. Case #1; unremarkable. B. Case #2; alterations include gain of 1q21.1q44, loss of 14q23.3q32.33 (including DICER1 and AKT1), and gain of chromosome 19 with a focal loss of 19q13.33q13.43 (including PPP2R1A, CACNG6, C19MC and TTYH1). C. Case #3; unremarkable. D. Case #4; unremarkable. [file 40478_2024_1746_MOESM2_ESM.docx]
